# Central leptin pathways in metabolic homeostasis

**DOI:** 10.1042/CS20257748

**Published:** 2025-11-17

**Authors:** Yuying Zhao, Connor Laule, Kamal Rahmouni

**Affiliations:** 1 Department of Neuroscience and Pharmacology, University of Iowa Carver College of Medicine, Iowa City, Iowa; 2 Department of Internal Medicine, University of Iowa Carver College of Medicine, Iowa City, Iowa, 52242; 3 Fraternal Order of Eagles Diabetes Research Center, University of Iowa Carver College of Medicine, Iowa City, Iowa; 4 Obesity Research and Education Initiative, University of Iowa Carver College of Medicine, Iowa City, Iowa; 5 Veterans Affairs Health Care System, Iowa City, Iowa

**Keywords:** energy balance, glucose homeostasis, hypothalamus, neural circuits, obesity

## Abstract

Obesity continues to be a major global health crisis, contributing to the rising prevalence of metabolic disorders such as type 2 diabetes, cardiovascular disease, and certain cancers. Central to the regulation of energy homeostasis is the adipocyte-derived hormone leptin, which serves as a key afferent signal to the central nervous system to suppress food intake, enhance energy expenditure, and maintain glucose balance. Since its discovery over three decades ago, a wealth of research has illuminated the molecular, cellular, and physiological mechanisms through which leptin exerts its metabolic effects. These foundational studies have delineated the neural circuits, particularly within the hypothalamus and brainstem, that integrate leptin signaling to co-ordinate complex metabolic responses. This review provides a comprehensive synthesis of the current understanding of leptin’s metabolic actions, with an emphasis on the intracellular signaling cascades that mediate leptin receptor activation. We also highlight the diverse neuronal populations and brain regions that contribute to leptin’s regulatory roles.

## Introduction

**Figure 1:**
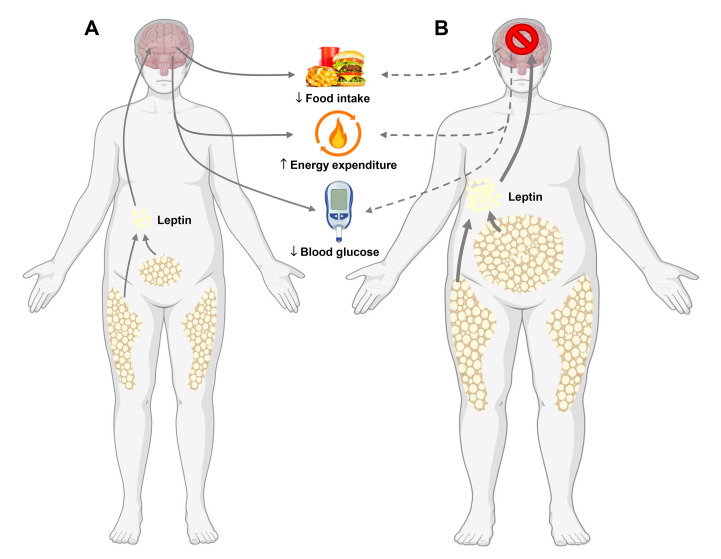
Leptin regulates energy and glucose homeostasis. **(A**) Leptin is a circulating endocrine factor produced by adipocytes. Leptin signals to the brain to reduce food intake, stimulate energy expenditure, and decrease systemic glucose levels. (**B**) In obesity, increased adiposity is accompanied by elevated circulating leptin levels, which promote leptin resistance and thereby impair leptin’s regulatory effects on appetite, energy expenditure, and glucose homeostasis.

Overweight and obesity are highly prevalent conditions affecting two-thirds of adults in the United States, which imposes an enormous economic burden and contributes to type 2 diabetes, cardiovascular disease, and certain cancers [1,2]. Over the past several decades, extensive research has implicated leptin as a key regulator of energy homeostasis and a central player in the pathophysiology of obesity. Leptin is an adipocyte-derived hormone secreted, in proportion to fat mass, into the bloodstream and signals in various tissues, particularly the central nervous system. Leptin activates its cognate leptin receptor (LepR) in target tissues to regulate metabolism, with the long form LepR (LepRb) being strongly implicated in leptin’s effect on energy balance and glucose metabolism [3]. Leptin signaling to the brain decreases appetite, stimulates metabolic rate, and lowers systemic glucose levels (Figure 1). In most cases of common obesity, circulating leptin levels are elevated, yet its anorexigenic and weight-reducing effects are blunted—a phenomenon known as leptin resistance. This paradox has significantly shaped our current understanding of metabolic regulation and the complex neuroendocrine mechanisms that perpetuate obesity. Ongoing research continues to investigate the molecular and cellular mechanisms driving leptin resistance, with the goal of identifying new therapeutic strategies to combat obesity and associated complications.

Absence of leptin or its receptor causes significant metabolic disorders such as obesity and type 2 diabetes. Indeed, mice harboring mutations in leptin (e.g., *ob/ob* mice) or LepRb (e.g., *db/db* mice) genes display severe hyperphagia, obesity, and insulin resistance. These mouse models have been valuable for elucidating molecular and cellular pathways that co-ordinate leptin’s metabolic actions.

Leptin resistance is a common phenomenon in obesity that renders the metabolic actions of leptin ineffective, even with high doses of exogenous leptin. As such, efforts to treat common obesity with leptin have failed. Recently, glucagon-like peptide-1 (GLP-1) analogs have set a new benchmark for anti-obesity treatment. Preclinical studies show that GLP-1 combination therapy with other endocrine analogs, such as leptin, is superior to GLP-1 monotherapy. Thus, understanding the basis for how leptin produces negative energy balance is important for designing multipronged weight loss regimens.

### Leptin receptor signaling

The interaction between leptin and its receptors is essential for the activation of downstream signaling pathways. The LepRb is classified as a member of the class I cytokine receptor family and is characterized by its transmembrane structure, which includes an extracellular ligand-binding domain, a transmembrane domain, and a cytoplasmic domain. Upon leptin binding to the LepRb, a series of intracellular downstream signaling pathways are initiated.

**Figure 2:**
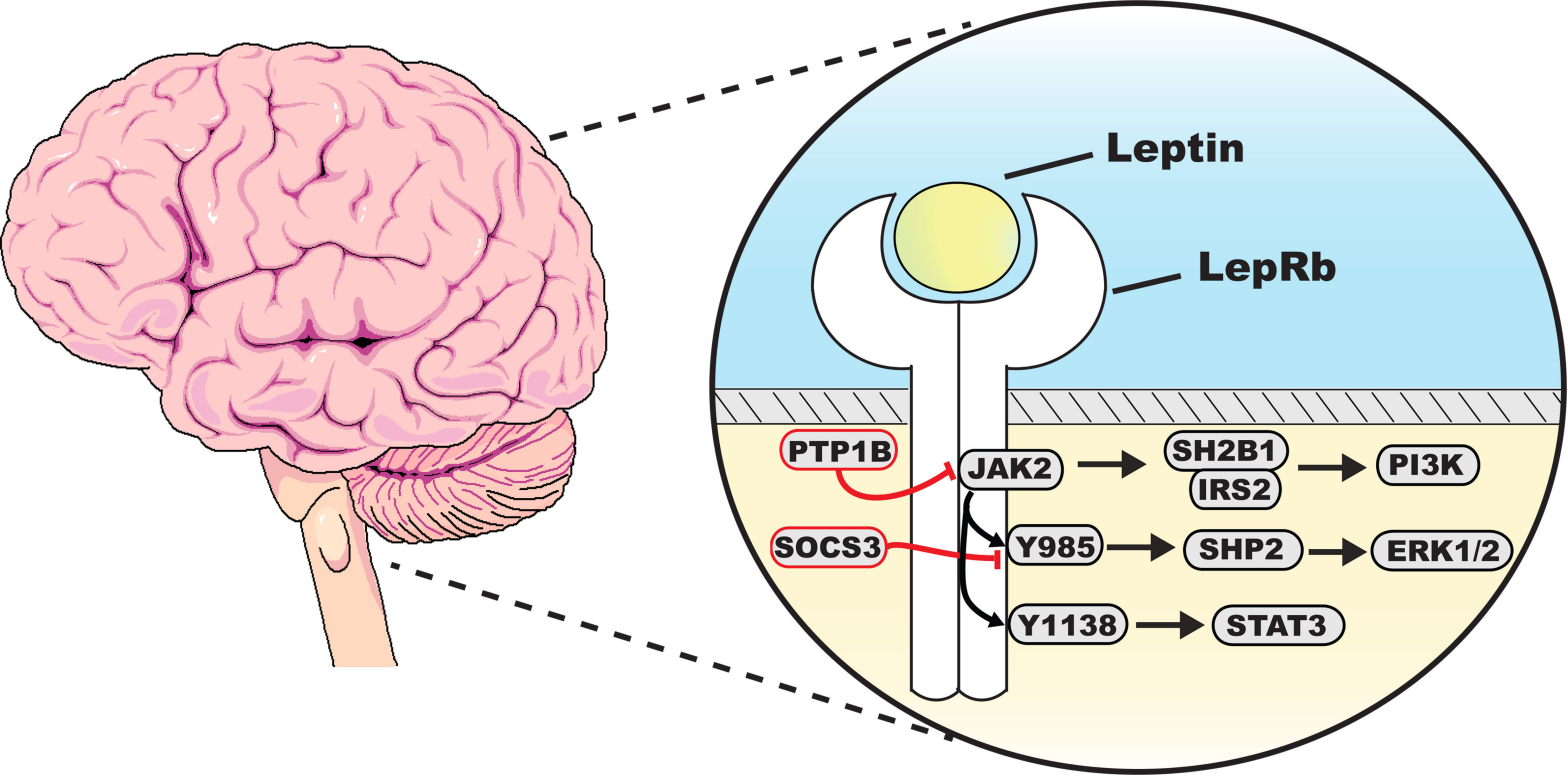
**Intracellular signaling pathways underlying leptin regulation of metabolism**. Leptin activation of the long signaling isoform of the leptin receptor (LepRb) engages multiple intracellular pathways. In the brain, downstream signaling proteins mediate leptin’s actions on metabolism. Negative feedback by PTP1b and SOCS3 overactivation are implicated in obesity-associated leptin resistance. ERK1/2, extracellular signal-regulated kinase 1 and 2; IRS2, insulin receptor substrate 2; JAK2, Janus kinase 2; PI3K, phosphatidylinositol-3-kinase; PTP1b, protein tyrosine phosphatase 1b; SH2B1, SH2 adaptor protein 1; SHP2, Src-homology 2 domain-containing phosphatase; SOCS3, suppressor of cytokine signaling 3; STAT3, signal transducer and activator of transcription 3; Y1138, tyrosine residue 1138; Y985, tyrosine residue 985.

Like many other cytokine receptors, LepRb necessitates the presence of a non-covalently associated kinase, specifically Janus kinase 2 (JAK2), for effective signal transduction. Upon leptin binding to the extracellular domain of the LepRb, a conformational change occurs, enabling the recruitment of JAK2. This allows JAK2 to undergo autophosphorylation, culminating in the stabilization of a LepRb-JAK2 complex. Activated JAK2 proceeds to phosphorylate key tyrosine residues on the cytoplasmic domain of the LepRb including Tyr985, Tyr1077, and Tyr1138, which provide loading sites for various signaling cascades (Figure 2).

### JAK2-STAT3/5/1 pathway

Following the activation of JAK2 by leptin, signal transducer and activator of transcription 3 (STAT3) is selectively recruited to the phosphorylated Tyr1138 on LepRb, where it undergoes further phosphorylation by JAK2. This phosphorylation event enables STAT3 to translocate to the nucleus, initiating the transcriptional programs of target genes integral to energy homeostasis. Numerous studies have established JAK2-STAT3 signaling as leptin’s major signaling pathway that regulates energy balance. Work conducted by Bates et al. found that mice with a loss-of-function mutation in LepRb Tyr1138 (LepRb^ser1138^) are obese with hyperphagia, reduced locomotor activity, and compromised cold tolerance [4,5]. This emphasizes the importance of Tyr1138 phosphorylation in mediating leptin’s action. Interestingly, these LepRb^ser1138^ mice displayed improved insulin resistance and glucose intolerance when compared with *db/db* mice, suggesting that other signaling pathways contribute to glucose homeostasis. Further findings by Bates and colleagues indicate that the observed physiological changes in LepRb^ser1138^ mice correlate with compromised thyroid function and reduced expression of uncoupling protein-1 (UCP-1) [4]. This evidence suggests that the thyroid hormone (TH) may contribute to leptin-mediated metabolic balance, specifically within peripheral tissues. A recent study has confirmed that exogenous leptin enhances the activity of type 2 deiodinase, an enzyme involved in thyroid hormone activation, thereby augmenting intramuscular triiodothyronine (T3) levels in skeletal muscles [6]. Moreover, selective STAT3 deletion in LepRb-expressing neurons induces hyperphagia and obesity, mirroring the phenotypes of LepRb Tyr1138 deficiency [7]. Conversely, STAT3 deficiency in defined neuronal populations such as those expressing proopiomelanocortin (POMC) or agouti-related peptide (AgRP) only modestly affects food intake and body weight [8,9]. Of note, STAT3 serves as a transcription activator for genes such as POMC, AgRP, and neuropeptide Y (NPY) [5,10,11], with evidence indicating that its removal in neurons reduces POMC expression, thereby underscoring a regulatory role [11].

In addition to STAT3, another member of the STAT family, STAT5, is phosphorylated in response to LepRb activation, enabling its nuclear translocation and transcriptional modulation of target genes. LepRb Tyr1077 functions as a dominant site for the recruitment of STAT5. However, LepRb-STAT5 appears to exert a relatively minor effect on energy balance. Although mutations in LepRb Tyr1077 associate with mild increases in adiposity, and STAT5 knockout mice developed late-onset obesity, recent findings suggest that STAT5 deletion in LepRb-expressing neurons does not cause obesity [12–14]. Additionally, ablation of STAT1, which is recruited to LepRb Tyr1138, in LepRb neurons failed to alter energy homeostasis [14]. Collectively, these observations identify STAT3 as the main mediator of leptin’s metabolic actions [15].

### SHP2-ERK signaling

Phosphorylation of Tyr985 on LepRb facilitates its interaction with the Src homology 2 (SH2) domain of protein tyrosine phosphatase 2 (SHP2). This activates the extracellular signal-related kinase 1/2 (ERK1/2) pathway, which is crucial for energy homeostasis [15,16]. Although deficiency in LepRb Tyr985 caused a lean phenotype despite the occurrence of late-onset obesity [17,18], SHP2 deletion in the forebrain promotes early-onset obesity [19]; and its deletion in POMC neurons leads to mild obesity [20,21]. On the other hand, activation of extracellular signal-regulated kinases 1 and 2 (ERK1/2) by leptin negatively regulates food intake and body weight [15]. Despite these apparent disparities, SHP2-ERK signaling remains essential for energy control. Mechanistically, this signaling can down-regulate JAK2-STAT3 through the binding of the SH2 domain of suppressor of cytokine signaling 3 (SOCS3) to LepRb Tyr985, contributing to leptin’s anti-obesity effects [22].

### SH2B1-PI3K pathway

The phosphoinositide 3-kinase (PI3K) pathway plays a significant role in leptin signaling by regulating food intake and energy expenditure. Activation of PI3K by the LepRb involves the recruitment of the insulin-receptor substrate 2 (IRS2) to JAK2 via the SH2 adaptor protein 1 (SH2B1) followed by IRS2 phosphorylation. Phosphorylated IRS2 then binds to the p85 subunit of PI3K, leading to its activation and accumulation of phosphatidylinositol 3,4,5-trisphosphate (PIP3) and subsequent activation of protein kinase B (also called AKT). Studies have demonstrated that mice with SH2B1 deficiency are obese with leptin resistance, hyperphagia, and diabetes [23,24]. Importantly, restoring neuronal SH2B1 in SH2B1 knockout mice rescued hyperphagia and obesity and protected from diet-induced weight gain and insulin resistance [25,26], indicating that neuronal SH2B1 is essential for controlling energy and glucose homeostasis. Furthermore, mice with IRS2 deficiency in the brain displayed increased adiposity and leptin resistance [27,28], confirming the importance of IRS for leptin signaling. While IRS2 deletion in LepRb neurons caused leptin resistance and obesity in mice [29], its ablation in POMC neurons did not significantly affect metabolic balance [28]. Despite this disparity, potentially stemming from variable LepRb expression in different brain regions or compensatory mechanisms from other pathways, SH2B1-IRS2 signaling significantly contributes to leptin action.

In the context of leptin signaling, activation of PI3K engages key downstream targets involved in metabolic homeostasis such as AKT and mechanistic target of rapamycin complex 1 (mTORC1). Intracerebroventricular (ICV) administration of PI3K inhibitors in mice blocks leptin’s anorexigenic effects [30,31]. Conversely, enhancement of PI3K signaling in LepRb neurons by deleting the negative regulator gene, *Pten*, decreased body weight and adiposity due to sympathetically mediated increase in metabolic rate without change in feeding behavior [32]. Both *in vitro* and *in vivo* studies demonstrate that PI3K inhibitors abrogate the depolarization of POMC neurons evoked by leptin [30,33]. The ablation of the PI3K regulatory subunit, p85, in POMC neurons disrupted leptin’s ability to decrease food intake [33]. Additionally, disruption of the PI3K catalytic subunit, p110β, in POMC neurons caused hyperphagia, increased adiposity, and hyperleptinemia, whereas PI3K p110β deletion in AgRP neurons did not lead to increased body weight [34]. Taken together, these data suggest that leptin recruits PI3K signaling to regulate metabolism.

### Negative regulation of LepRb signaling

Inhibition of LepRb intracellular signaling by JAK2 dephosphorylation is a potential contributor to leptin resistance in obesity. Increased LepRb negative feedback blunts leptin’s ability to activate divergent signaling cascades downstream of JAK2. In support of this, leptin stimulation of STAT3 and ERK1/2 is impaired in the arcuate nucleus (ARC) of the hypothalamus in mice with diet-induced obesity (DIO) [35,36]

Notably, SOCS3 functions as a negative feedback regulator of the LepRb. Leptin activation of STAT3 induces the transcription of several genes, including SOCS3. In turn, SOCS3 inhibits JAK2 and diminishes LepRb signaling via interactions with Y985 [22]. Heterozygous SOCS3 deficiency results in enhanced leptin sensitivity and improved hypothalamic LepRb signaling in response to exogenous leptin; importantly, it protects against DIO [37]. SOCS3 deficiency in the brain also potentiates leptin sensitivity and resistance to DIO [38]. Strikingly, SOCS3 deletion in LepRb neurons fails to protect against DIO, but it does protect from diet-induced insulin resistance [39]. Consistently, SOCS3 overexpression in POMC neurons leads to leptin resistance and mild obesity [40]. Mice bearing SOCS3 deletion in AgRP neurons also displayed leptin resistance, however, without changes in body weight [41]. These discrepancies in body weight evoked by SOCS3 deficiency from different neurons may be due to their heterogeneity or compensatory mechanisms. Of note, *SOCS3* levels in the ARC are elevated in DIO [35,42].

Leptin signaling is also negatively modulated by protein tyrosine phosphatase 1B (PTP1B). PTP1B suppresses leptin-stimulated phosphorylation of JAK2 and STAT3 in a dose-dependent fashion [43]. Mice lacking PTP1B in the whole body or in the brain are hypersensitive to leptin and resistant to obesity [44,45]. Strikingly, mice with the LepRb deletion from neurons displayed a leaner body weight compared with mice with its ablation in whole body, suggesting that PTP1B signaling in neurons plays a predominant role in negatively regulating leptin’s metabolic actions [46]. Indeed, *in situ* hybridization unveiled that PTP1B is highly expressed in hypothalamic LepRb neurons including the ARC, ventromedial hypothalamus (VMH), and dorsomedial hypothalamus (DMH) [43]. On the other hand, the adipocyte-specific deletion of PTP1B results in weight gain, whereas deletions of PTP1B from the liver or skeletal muscle failed to alter body weight [44]. Collectively, these findings underscore the importance of PTP1B in leptin’s control of energy balance.

It is worth noting that, beyond SOCS3 and PTP1B, several additional molecules and cellular mechanisms have been implicated as negative regulators of LepRb signaling and may contribute to the development of leptin resistance in obesity. This includes PTP non-receptor type 2 (PTPN2), PTPε, exchange protein directly activated by cyclic AMP 1 (EPAC1), and ER stress [47].

### Other determinants of leptin signaling pathways

#### SHP2-MAPKs

Increasing evidence shows the involvement of the AMP-activated protein kinase (AMPK) in leptin regulation of energy homeostasis [48–50]. For example, leptin has been found to inhibit AMPK activity in hypothalamic neurons to reduce appetite and lower body weight [50]. On the other hand, leptin has been shown to activate AMPK and decrease acetyl-CoA carboxylase (ACC) activity, thereby facilitating fatty acid oxidation in skeletal muscle [51]. These data suggest that leptin has tissue-specific effects on AMPK to influence metabolic homeostasis.

#### BBSome and cilia

 Primary cilia, antenna-like sensory organelles protruding from the surface of most vertebrate cell types including neurons, have been implicated in hyperphagia-induced obesity and leptin resistance [52]. The size and the number of hypothalamic cilia are reduced in *ob/ob* and *db/db* mice compared with the age-matched C57 mice, with leptin infusion in ob/ob mice increasing the length of neural cilia accompanied with improved leptin sensitivity [52]. This process has been demonstrated to be regulated by the JAK2-PI3K pathway and can be down-regulated by PTEN [52,53]. Additionally, mice with cilia dysfunction, such as adenylate cyclase 3 (AC3) deletion, displayed leptin resistance and obesity, indicating cilia involvement in leptin signaling [54]. Particularly, mice bearing a mutation in cilia related protein retinitis pigmentosa GTPase regulator-interacting protein-1 like protein (*Rpgrip1l*) show decreased cilia frequency in the hypothalamus and dampened leptin sensitivity through STAT3 suppression [55]. These findings suggest that leptin regulates body weight by modulating cilia length.

Generally, cilia membranes are equipped with various proteins, including G protein-coupled receptors, to sense extracellular signals. Substantial evidence demonstrates the importance of the BBSome, a protein complex of eight conserved Bardet-Biedl syndrome (BBS) proteins, in transporting receptors to and from the cilia [56–58]. In addition, the BBSome has emerged as an essential player in trafficking receptors to the plasma membrane, including LepRb [59,60]. A critical component of the BBSome (BBS1) was found to physically interact with the C-terminal cytoplasmic segment of LepRb. BBSome deficiency reduces LepRb surface expression, which impairs leptin’s effects and probably contributes to obesity in BBS individuals. Indeed, mice bearing the *Bbs1* ^M390R^ mutant or with *Bbs1* deletion in specific neurons are leptin resistant, hyperphagic, and obese [59,61–63]. However, BBSome involvement in the transport of LepRb is independent of cilia since mice with cilia removal through *Ift88* (intraflagellar transport 88) gene deletion did not develop leptin resistance [59]. The difference in phenotypes between AC3 knockout and Ift88 knockout mice may result from the relatively narrow effects of AC3-evoked cAMP signaling compared with the large number of signaling pathways affected by cilia dysfunction through *Ift88* gene deletion in the neurons. BBS17 was also found to regulate leptin receptor signaling by activating STAT3 in the hypothalamus [64]. A recent study showed that BBS10, a key component of the BBS chaperonin complex, is required for BBSome formation and stabilizes the LepRb by reducing its degradation or promoting its translation [65].

## Central leptin signaling in energy homeostasis

The obesity phenotype in leptin- and LepRb-deficient mice is largely recapitulated by LepRb deletion specifically from neurons [66] highlighting the importance of the brain in mediating the metabolic effects of leptin. This has incited research into the neural underpinnings of leptin’s metabolic actions, which has identified several important brain regions and neuronal subsets.

### Neuronal substrate of appetite regulation by leptin

**Figure 3:**
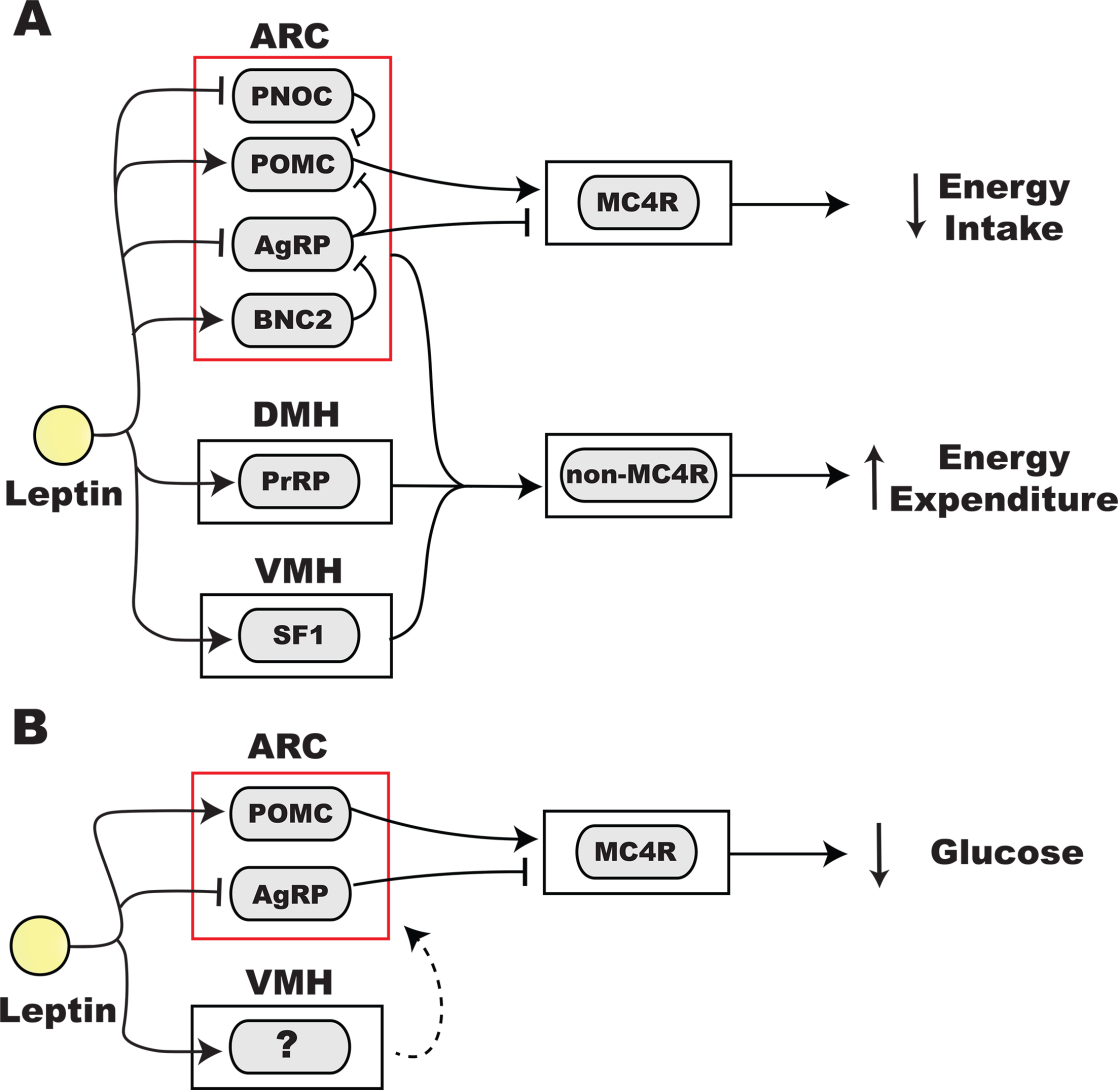
Neural circuits involved in the metabolic effects of leptin. **(A**) Leptin influences energy balance by decreasing energy intake and increasing energy output. Leptin signaling to ARC neurons promotes activation of downstream MC4R neurons which reduces food intake. Leptin activation of neurons in the ARC, DMH, and VMH are integrated by downstream non-MC4R neurons to boost energy expenditure. (**B**) Leptin directly acts in the ARC and VMH, resulting in the activation of downstream MC4R cells to decrease systemic glucose. Red box indicates the brain region (ARC) where leptin resistance occurs in obesity. AgRP, agouti-related peptide; ARC, arcuate nucleus of the hypothalamus; BNC2, basonuclin 2; DMH, dorsomedial hypothalamus; MC4R, melanocortin-4 receptor; PNOC, prepronociceptin; POMC, proopiomelanocortin; PrRP, prolactin-releasing peptide; SF1, steroidogenic factor 1; VMH, ventromedial hypothalamus.

Early *in situ* hybridization mapping found robust LepRb expression in the hypothalamus and other regions involved in feeding behavior [67–69]. Direct brain injection of leptin reduces food intake supporting a central mechanism of action [68,70]. These findings have stimulated research into the cell types and circuits that orchestrate leptin’s action on appetite. Leptin primarily regulates energy intake by modulating ARC microcircuits and melanocortinergic output (Figure 3A).

Leptin microinjection experiments identified the ARC as a primary region for leptin to suppress feeding [71]. This led to an investigation into leptin-sensitive neuron populations in the ARC. AgRP and POMC cell types are established as opposing appetite regulators. Activation of AgRP neurons increases food intake, whereas activation of POMC neurons decreases feeding [72]. Leptin simultaneously inhibits AgRP neurons and activates POMC cells to reduce food intake [73,74]. Since POMC neurons receive inhibitory synaptic input from AgRP cells, leptin stimulation of POMC neurons probably occurs through both direct signaling and indirect synaptic effects [73,75]. Distinct leptin-sensitive ARC neurons that express gamma-aminobutyric acid (GABA) have recently emerged as critical regulators of AgRP and POMC neuronal activity. Inhibitory ARC neurons characterized by basonuclin 2 (BNC2) expression were shown to monosynaptically inhibit AgRP cells, decrease feeding, and are excited by leptin [76]. These findings suggest that leptin activation of BNC2 neurons reduces appetite by inhibiting AgRP neurons. Another GABAergic ARC population, defined by prepronociceptin (PNOC) expression, was found to monosynaptically inhibit POMC cells, increase food intake, and be inhibited by leptin [77]. These results suggest that leptin silences PNOC neurons, leading to increased POMC neuronal activity to reduce appetite [78]. Leptin-sensitive excitatory ARC neurons have also been implicated in appetite regulation, albeit with less molecular resolution. Glutamatergic neurons in the caudal ARC provide synaptic input to POMC neurons [79]. These cells may regulate appetite by integrating hormonal inputs such as leptin and the anorectic hormone, cholecystokinin. Further work is required to clarify genetic markers for excitatory ARC population and assess their functional role. Collectively, these findings reveal that leptin modulates appetite by simultaneously signaling to AgRP and POMC neurons and by regulating upstream local ARC microcircuits. It will be important to understand how obesogenic diets and leptin resistance reshapes these ARC pathways, leading to hyperphagia and obesity.

Conditional knockout experiments have validated an important role for leptin signaling in the ARC. Constitutive deletion of the LepRb from either AgRP, POMC, BNC2, or PNOC neurons moderately increases body weight without affecting food intake [76,78,80–82]. Moreover, constitutive knockout of the LepRb from both POMC and AgRP neurons in the same animals produced a similar phenotype [80]. A major limitation from this approach is developmental compensation, which could explain the moderate phenotypes. In line with this, CRISPR-mediated LepRb deletion from AgRP cells in adult mice phenocopied the severe hyperphagia and obesity observed in global LepRb deficient animals [83]. Consistent with the ARC as a primary regulator of leptin’s effect on appetite, deletion or knockdown of the LepR from other brain regions has limited effects on feeding [84–88].

AgRP and POMC neurons produce melanocortin peptides that inhibit and activate the melanocortin-4 receptor (MC4R), respectively [89–91]. Expression of MC4R is required for the POMC-derived peptide, α-melanocyte hormone (α-MSH), to reduce food intake [92]. Several lines of evidence support a role for the melanocortin system in leptin’s regulation of appetite and body weight. Humans and rodents with genetic MC4R deficiency exhibit hyperphagic obesity [93–98]. Pharmacological inhibition and activation of brain MC4R elicits hunger and satiety, respectively [99]. Furthermore, pharmacological blockade or genetic deletion of MC4R prevents the anorectic and weight-reducing actions of leptin [100,101]. In line with a role for the leptin-melanocortin system in energy homeostasis, individuals with genetic defects in leptin signaling, POMC, or MC4R present with increased appetite and obesity [102]. The notion that MC4R is downstream of leptin targets is supported by the effectiveness of MC4R agonism to reduce hyperphagia and obesity in leptin-deficient individuals [103].

MC4R neurons are widely distributed throughout the brain with high expression in the paraventricular nucleus of the hypothalamus (PVH) [104]. The PVH plays an important role in appetite control and is widely considered as the second-order structure mediating leptin’s satiety effect. Both AgRP and POMC neurons innervate the PVH, and this region contains a large population of MC4R-expressing neurons. PVH MC4R neurons are activated by α-MSH and inhibited by AgRP [105]. A role for PVH MC4R cells in appetite is supported by studies showing that hyperphagia in MC4R null mice is completely reversed by selective re-expression of this receptor in the PVH [106,107]. Furthermore, modulation of AgRP, POMC, and PVH MC4R neuron activity produces effects on food intake in line with this model [72,108,109].

MC4R neurons are also present in other brain regions involved in appetite regulation, such as the VMH and lateral hypothalamus, suggesting that divergent outputs from ARC melanocortinergic neurons may mediate leptin’s effects on appetite [110,111]. Systematic assessment with contemporary genetic and neuroscience approaches are needed to define the MC4R neurons that mediate leptin’s actions on appetite.

### Leptin’s central actions on energy expenditure

In addition to decreasing food intake, leptin promotes negative energy balance by increasing energy expenditure and lipid metabolism [112–114]. Brain infusion of leptin promotes fat oxidation and prevents adaptive reductions in energy expenditure caused by reducing energy intake [115,116]. Exogenous leptin profoundly lowers circulating triglyceride levels in individuals with severe lipodystrophy independent of food intake [117,118]. Preclinical experiments suggest this occurs through leptin’s actions in the brain and presumably the autonomic nervous system [119]. Given the beneficial effects of leptin to promote negative energy balance, the neural mechanisms underlying lipid disposal and energy expenditure have been investigated. In contrast to its effects on appetite, leptin apparently signals through multiple brain regions to stimulate metabolism independent of the melanocortin system (Figure 3A).

Fat stored in white adipose tissue is mobilized by sympathetic nerve activation to meet energy demands [120]. Excess lipid stores are communicated to the brain via leptin that subsequently engages the sympathetic nervous system (SNS) to induce lipolysis and fat mobilization [121]. Circulating free fatty acids are oxidized by brown adipose tissue (BAT), which is primarily regulated by the SNS [15,122]. Leptin’s sympathoexcitatory actions regulate adipose tissue gene programs to promote lipid utilization through the SNS [123–125]. Many neuronal populations anatomically connected to adipose tissue express the LepRb, providing a blueprint for leptin’s site of action [67,126–129].

The ARC appears to be a primary site for leptin-induced energy expenditure. Virally mediated deletion of the ARC LepRb in adult mice abolishes leptin-induced BAT sympathetic activation [130]. Subsequent experiments in mice with constitutive LepRb deletion suggest that activation of BAT sympathetic nerves occurs through leptin’s actions on AgRP and POMC neurons [82]. Moreover, LepRb expression on AgRP and POMC neurons facilitates leptin-induced adipose tissue sympathetic innervation [131]. Consistent with the excitatory effects of leptin on POMC neurons, chemogenetic POMC neuron activation promotes lipid metabolism [132]. On the other hand, leptin inhibition of AgRP neurons probably promotes energy expenditure since AgRP neuron activation decreases thermogenesis and lipid utilization [133,134].

Since central MC4R activation increases energy expenditure and BAT activity, a role for ARC melanocortin output has been speculated [135–137]. However, MC4R antagonism failed to attenuate leptin-induced increase in BAT temperature and BAT SNS activity, indicating non-melanocortin pathways mediate leptin’s stimulation of energy expenditure [137,138]. One possibility is that leptin silencing of AgRP neurons reduces NPY output by these neurons, which is expected to decrease energy expenditure [139–143]. Owing to the diverse projection profiles of AgRP and POMC neurons, studies employing contemporary genetic and neural circuit techniques are required to delineate the pathways that co-ordinate leptin’s effect on energy expenditure [110].

Leptin signaling to other hypothalamic structures has also been implicated in energy expenditure. For instance, leptin administration into the DMH raises body temperature to normal levels in leptin-deficient mice [86,138]. Deletion of LepRb from DMH neurons lowers energy expenditure and drives weight gain, largely independent of food intake [86]. The LepRb on DMH neurons expressing prolactin-releasing peptide (PrRP) is required for leptin induction of thermogenesis but not satiety [144]. Electrophysiological recordings showed that leptin activates DMH LepRb+ neurons [145] and chemogenetic activation of these neurons promotes thermogenesis and energy expenditure [86].

A role for leptin signaling in the VMH in the control of energy expenditure is supported by the finding that LepRb deletion from this region increases adiposity and plasma triglycerides independent of food intake [84,85]. Furthermore, electrophysiological recordings show that leptin activates VMH neurons and chemogenetic activation of the VMH increases energy expenditure [85,146]. Although studies are limited, the supraoptic nucleus, nucleus raphe pallidus, and preoptic nucleus also appear to mediate leptin’s effect on sympathetic output to adipose tissue [147–149].

LepRb expression in interoceptive sensory neurons may also co-ordinate leptin’s effect on energy expenditure [150–152]. For example, LepRb deletion from vagal afferents reduces lipogenesis [153]. Another possibility is leptin acts on adipose-innervating spinal afferents which were recently shown to regulate lipogenic and thermogenic transcriptional programs [154]. In line with this, leptin administration to white adipose tissue activates local afferents and sympathetic outflow to fat pads and other organs [155–157]. These findings suggest a model where leptin’s paracrine actions engage a feedforward autonomic circuit that promotes sympathetic outflow and fat metabolism. However, more work is needed to determine the physiological relevance of leptin signaling in spinal afferents.

### Neural underpinnings for leptin’s regulation of glucose metabolism

Leptin has been identified as a potentiator of peripheral glucose utilization. Early studies demonstrated that hyperglycemia in leptin-deficient mice could be restored with exogenous leptin treatment [54,112,158]. While the anorectic and weight-lowering effect of leptin might indirectly lower glucose in these mice, several lines of evidence indicate that leptin has direct actions on glucose clearance. First, leptin’s lowering of glucose precedes fat mass reductions in leptin-deficient mice [159]. Second, re-expression of the LepRb in various neuronal populations had no or limited effects on food intake and body weight but still profoundly lowered glucose relative to leptin null controls [160–163]. Third, in lean wildtype mice, central administration of leptin increased glucose uptake in peripheral tissues without changing body weight [164]. Consistently, brain action of leptin lowers glucose in a preclinical model of lean lipodystrophy [119]. Moreover, in lipodystrophic patients, leptin reduces glucose independent of food intake [118]. Similar to its regulation of appetite, leptin signaling in the ARC promotes glucose clearance through the melanocortin pathway; however, the VMH also appears to play an important role (Figure 3B).

The finding that leptin enhances hypothalamic insulin sensitivity led to speculation that insulin plays a mechanistic role in leptin’s effects on glucose. However, leptin action in the brain was found to restore euglycemia in mouse models of insulin deficiency, suggesting this is independent of insulin [165–167]. These reductions in systemic glucose were associated with increased glucose uptake by peripheral organs [166]. It was found that LepRb re-expression in GABAergic and POMC neurons or leptin infusion into the VMH is sufficient for leptin to lower glucose in type 1 diabetic mice [160,161].

The increase in glucose uptake in peripheral organs evoked by central leptin administration points to the autonomic nervous system as a mediator of circulating glucose clearance [164]. This is supported by the ability of leptin to increase sympathetic and parasympathetic nerve activity to organs involved in glucose metabolism including the liver, adipose tissue, and skeletal muscle [82,168]. The ARC and VMH are major pre-autonomic nuclei implicated in leptin’s regulation of glucose. LepRb re-expression in the ARC of adult mice lowers glucose [160]. Re-expression of the LepRb in POMC or AgRP neurons is sufficient for leptin to lower circulating glucose [161–163]. NPY or GABA released from AgRP neurons is not required for the glucose-lowering effects of leptin, suggesting the involvement of other systems such as the melanocortin receptors. Consistently, leptin’s ability to lower circulating glucose and elevate peripheral glucose uptake was found to require the MC4Rs [163,169].

Microinjections of leptin into the VMH evoke sympathetic activation to promote glucose uptake by peripheral tissues [170,171]. Central melanocortin signaling is required for leptin signaling in the VMH to increase peripheral glucose uptake [169]. Since VMH neurons are non-melanocortinergic, this suggests a relay through ARC neurons may be involved. Consistently, leptin injection into the VMH increases ARC c-Fos expression, but not STAT3 activation, indicating a downstream effect of leptin-VMH signaling on ARC neuron activity [169]. The VMH and PVH may contain MC4R neurons that mediate leptin’s effect since MC4R agonist injection into either brain region promotes peripheral glucose uptake [169]. Strikingly, VMH MC4R activation promotes glucose uptake by both BAT and muscle, whereas PVH MC4R stimulation only influences BAT glucose uptake [169]. Thus, leptin may engage distinct melanocortin populations to regulate glucose metabolism in an organ-specific manner. The precise pre-autonomic circuits underlying leptin regulation of glucose warrant future investigation.

## Conclusions and perspectives

****Three decades have passed since the discovery of leptin, and our understanding of its multifaceted roles in metabolic homeostasis has significantly advanced. Recent findings have defined new neuronal cell types that orchestrate leptin’s effects on energy balance and glucose homeostasis. The identification of novel neuronal mediators such as BNC2 and PNOC neurons in the ARC offers new avenues for defining and possibly manipulating leptin’s impact on body weight [76,78,172]. Despite these advances, the persistent challenge of leptin resistance in obesity underscores the need for continued research into the molecular and cellular mechanisms that impair leptin responsiveness.

 Leptin replacement therapy has shown remarkable success in individuals with congenital leptin deficiency and lipodystrophy, leading to significant weight loss in leptin deficient patients, improved glycemic control, and decreased triglyceride levels [117]. In these cases, leptin effectively restores energy homeostasis and ameliorates metabolic abnormalities associated with severe leptin deficiency. However, the application of exogenous leptin as a monotherapy for common obesity, where circulating leptin levels are often elevated, is ineffective due to leptin resistance [173,174].

Leptin resistance is a major impediment to effective weight loss strategies, and the cause of leptin resistance remains elusive. Lowering leptin levels in obesity can promote leptin sensitivity [173,175]. The reduction in leptin by anti-obesity medications, such as GLP-1 agonists, contributes to their beneficial metabolic effects [175]. Consistently, preventing rapid leptin surges after anti-obesity treatment cessation delayed weight regain and glucose dysregulation [175]. This conceptual shift, focusing on partial leptin reduction to overcome hyperleptinemia-induced resistance, opens new therapeutic avenues for the managment of obesity and type 2 diabetes.

The limitations of leptin monotherapy in obesity have spurred interest in combination treatments, particularly those involving GLP-1 receptor agonists. GLP-1 receptor agonists are a highly effective class of satiety medications that promote weight loss and improve glucose regulation [176,177]. Given that leptin and GLP-1 are both anorectic hormones, their combination has been explored for weight loss in preclinical models [178]. Co-administration of GLP-1 and leptin significantly decreases food intake in rats [178]. While GLP-1 receptor agonists alone are effective for weight loss, combining them with leptin has demonstrated additive effects, leading to greater reductions in food intake [179,180]. This may occur through amplification of shared signaling pathways and/or by engaging converging satiety circuits. Studies in DIO mice have shown that GLP-1/glucagon co-agonism can restore leptin responsiveness [181]. Furthermore, combination treatment with leptin and a GLP-1 receptor agonist has shown beneficial effects on glucose metabolism in insulin-dependent diabetic mice [182]. Interestingly, GLP-1 therapy can attenuate reductions in plasma leptin during weight loss maintenance, potentially contributing to sustained weight loss by preventing the leptin drop that often signals weight regain [183].

Although LepRb neurons in the brainstem respond to gut signals that synergize with leptin to suppress food intake, and GLP-1 brainstem cells represent a subset of these cells, the activation of LepRb in hindbrain neurons evokes stronger and more durable food intake suppression independent of GLP-1 signaling [184]. This suggests that GLP-1-independent pathways, likely involving other neurotransmitters within LepRb neurons, play an important role in feeding suppression by the brainstem. This interplay illustrates the potential for multihormone combination therapies to boost weight loss and potentially overcome acquired resistance during treatment [184]. Future research should focus on optimizing the doses and ratios of leptin in combination therapies, understanding the molecular and circuit mechanisms driving leptin resistance, and defining the neural pathways that actuate leptin’s favorable metabolic effects. Furthermore, anti-obesity treatment is only possible by reducing appetite, with therapeutic stimulation of energy expenditure completely lacking. Identifying leptin pathways that stimulate metabolism may lead to novel targets for weight loss.

Directly targeting pathways that contribute to leptin resistance also holds potential for treating obesity. Recently, increased mTOR activity in POMC neurons was found to cause leptin resistance, and pharmacological mTOR inhibition restored leptin sensitivity, resulting in weight loss [185]. Additionally, during energy surplus, NPY released in the ARC acts on POMC neurons to impair leptin sensitivity and promote weight gain [186]. These findings provide novel molecular components of POMC neurons that may be targeted to combat leptin resistance in obesity. Chronic activation of ARC GABAergic neurons that lack LepRb promotes obesity despite intact leptin signaling, while their inhibition prevents and reverses DIO, revealing a novel mechanism of leptin resistance [187]. Clarifying the underlying mechanism(s) will be important for understanding how signals from leptin-sensitive and leptin-insensitive neurons interact to regulate metabolic homeostasis and to identify new anti-obesity targets. Promoting leptin transport from the periphery into the brain holds potential for restoring leptin sensitivity to improve metabolic health. Tanycytes located in the median eminence transport circulating leptin into the hypothalamus where it can act on target neurons and exert metabolic effects [188–190]. It is speculated that impaired transport of leptin by tanycytes contributes to leptin resistance [191]. Leptin transcytosis by tanycytes requires activation of both the LepR and the endothelial growth factor receptor on tanycytes [188,189]. Strikingly, leptin transcytosis is impaired in mice with DIO and was found to be restored by endothelial growth factor treatment [188]. However, the presence of LepR in tanycytes and its role in transporting leptin to hypothalamic neurons is controversial [192]. Additionally, leptin resistance in DIO appears to occur in the ARC but not in other brain regions containing LepRb + neurons such as the DMH and VMH [35,36,42,193] (Figure 3). Considering that DIO up-regulates SOCS3 in the ARC, but not in the DMH or VMH, it appears that cell-autonomous mechanisms also contribute to leptin resistance [35,42]. These collective findings warrant further investigation into approaches that promote leptin transport and cellular LepRb signaling to overcome leptin resistance in obesity.

Critical questions also remain regarding how leptin’s metabolic effects are modulated by sex, developmental stage, nutritional status, and interaction with other hormonal and neural signals. Addressing these knowledge gaps is essential for the development of targeted and effective anti-obesity therapies. Thus, elucidating the molecular and neural underpinnings of leptin action will inform future strategies for harnessing leptin pathways to combat obesity and its related complications.

## Open Access

This article has been published open access under our Subscribe to Open programme, made possible through the support of our subscribing institutions, learn more here: https://portlandpress.com/pages/open_access_options_and_prices#conditional

## Abbreviations

AC3: adenylate cyclase 3
ACC: acetyl-CoA carboxylase
AKT: protein kinase B
AMPK: AMP-activated protein kinase
ARC: arcuate nucleus
AgRP: agouti-related peptide
BAT: brown adipose tissue
BBS: Bardet-Biedl syndrome
BNC2: basonuclin 2
DIO: diet-induced obesity
DMH: dorsomedial hypothalamus
EPAC1: exchange protein directly activated by cyclic AMP 1
ERK1/2: extracellular signal-regulated kinases 1 and 2
GABA: gamma-aminobutyric acid
GLP-1: glucagon-like peptide-1
IRS2: insulin-receptor substrate 2
Ift88: intraflagellar transport 88
JAK2: Janus kinase 2
LepR: leptin receptor
LepRb: long signaling form of the LepR
MC4R: melanocortin-4 receptor
NPY: neuropeptide Y
PI3K: phosphoinositide 3-kinase
PIP3: phosphatidylinositol 3,4,5-trisphosphate
PNOC: prepronociceptin
POMC: proopiomelanocortin
PTP1B: protein tyrosine phosphatase 1B
PTPN2: PTP non-receptor type 2
PVH: paraventricular nucleus of the hypothalamus
PrRP: prolactin-releasing peptide
Rpgrip1l: regulator-interacting protein-1 like protein
SH2: Src homology 2
SH2B1: SH2 adaptor protein 1
SHP2: SH2 protein tyrosine phosphatase 2
SNS: sympathetic nervous system
SOCS3: suppressor of cytokine signaling 3
STAT1/3/5: signal transducer and activator of transcription 1, 3, or 5
TH: thyroid hormone
UCP-1: uncoupling protein-1
VMH: ventromedial hypothalamus
mTOR: mechanistic target of rapamycin
mTORC1: mTOR complex 1
